# Prevalence and Geographic Distribution of Obstetrician-Gynecologists Who Treat Medicaid Enrollees and Are Trained to Prescribe Buprenorphine

**DOI:** 10.1001/jamanetworkopen.2020.29043

**Published:** 2020-12-11

**Authors:** Max Jordan Nguemeni Tiako, Jennifer Culhane, Eugenia South, Sindhu K. Srinivas, Zachary F. Meisel

**Affiliations:** 1 Medical student, Yale School of Medicine, New Haven, Connecticut; 2 Center for Emergency Care and Policy Research, Department of Emergency Medicine, Perelman School of Medicine, University of Pennsylvania, Philadelphia; 3 Urban Health Lab, University of Pennsylvania Perelman School of Medicine, Philadelphia; 4 Department of Obstetrics, Gynecology and Reproductive Sciences, Yale School of Medicine, New Haven, Connecticut; 5 Leonard Davis Institute of Health Economics, University of Pennsylvania, Philadelphia; 6 Department of Obstetrics and Gynecology, Perelman School of Medicine, University of Pennsylvania, Philadelphia; 7 Center for Health Economics of Treatment Interventions for Substance Use Disorder, HCV, and HIV, University of Pennsylvania, Philadelphia

## Abstract

**Importance:**

The incidence of opioid use during pregnancy is increasing, and drug overdoses are a leading cause of postpartum mortality. Most women who are pregnant do not receive medications for treatment of opioid use disorder, despite the mortality benefit that these agents confer. Furthermore, buprenorphine is associated with milder symptoms of neonatal abstinence syndrome (NAS) compared with methadone.

**Objective:**

To describe the prevalence and geographic distribution across the US of obstetrician-gynecologists who can prescribe buprenorphine (henceforth described as X-waivered) in 2019.

**Design, Setting, and Participants:**

A cross-sectional, nationwide study linking physician-specific data to county- and state-level data was conducted from September 1, 2019, to March 31, 2020. Data were obtained on 31 211 obstetrician-gynecologists who accept Medicaid insurance through the Centers for Medicare & Medicaid Services Physician Compare data set and linked to the Drug Addiction Treatment Act buprenorphine-waived clinician list.

**Exposures:**

State-level NAS incidence and county-level uninsured rates and rurality.

**Main Outcomes and Measures:**

Prevalence and geographic distribution of obstetrician-gynecologists who are trained to prescribe buprenorphine.

**Results:**

Among the 31 211 identified obstetrician-gynecologists, 18 710 (59.9%) were women. Most had hospital privileges (23 236 [74.4%]) and worked in metropolitan counties (28 613 [91.7%]). Only 560 of the identified obstetrician-gynecologists (1.8%) were X-waivered. Obstetrician-gynecologists in counties with fewer than 5% uninsured residents had nearly twice the odds of being X-waivered (adjusted odds ratio [aOR], 1.59; 95% CI, 1.04-2.44; *P* = .04) compared with those in counties with greater than 15% uninsured residents. Compared with those located in metropolitan counties, obstetrician-gynecologists in suburban counties (eg, urban population of ≥20 000 and adjacent to a metropolitan area) were more likely to be X-waivered (aOR, 1.85; 95% CI, 1.26-2.71; *P* = .002). Compared with states with an NAS rate of 5 per 1000 births or less, obstetrician-gynecologists in states with an NAS rate of 15 per 1000 births or greater had nearly 5 times the odds of being X-waivered (aOR, 4.94; 95% CI, 3.60-6.77; *P* < .001). Obstetrician-gynecologists without hospital privileges were more likely to be X-waivered (aOR, 1.32; 95% CI, 1.08-1.61; *P* = .007).

**Conclusions and Relevance:**

Fewer than 2% of obstetrician-gynecologists who accept Medicaid are able to prescribe buprenorphine, and their geographic distribution appears to be skewed in favor of suburban counties. This finding suggests that there is an opportunity for health systems and professional societies to incentivize X-waiver trainings among obstetrician-gynecologists to increase patients’ access to buprenorphine, especially during pregnancy.

## Key Points

**Question:**

What is the geographic distribution across the US and prevalence of Medicaid-claimant obstetrician-gynecologists who are trained to prescribe buprenorphine?

**Findings:**

In this cross-sectional study including 31 211 Medicaid-claimant obstetrician-gynecologists, fewer than 2% were trained to prescribe buprenorphine. In addition, these physicians were more likely to work in primarily suburban counties.

**Meaning:**

Results of this study suggest that an opportunity exists for obstetrician-gynecologists to contribute to expanding the workforce of clinicians who can prescribe buprenorphine to further address the opioid epidemic’s association with maternal and infant morbidity and mortality.

## Teaser

This cross-sectional study examines the prevalence and geographic distribution within the US of obstetrician-gynecologists who are able to prescribe buprenorphine for women who are pregnant.

## Introduction

The US opioid epidemic has impacted women who are pregnant at high rates. Although the overall proportion of admissions for drug treatment episodes among women who are pregnant has remained stable at 4%, the proportion reporting prescription opioids as the primary substance used increased substantially, from 1% to 19%, between 1992 and 2012.^1^ The rate of neonatal intensive care unit admissions owing to neonatal abstinence syndrome (NAS) has increased from 1.2 to 8 per 1000 births between 2000 and 2014 nationally, consistent with increased rates of opioid use during pregnancy.^2,3^ In states disproportionately impacted by opioid use disorder (OUD) such as West Virginia and Maine, NAS rates are as high as 50.6 per 1000 births (West Virginia) and 80 per 1000 births (Maine).

Standard of care for OUD in pregnancy includes pharmacotherapy.^4^ Per the American College of Obstetrics and Gynecology’s guidelines, the opioid agonist medications methadone and buprenorphine are the recommended treatments for OUD in pregnancy.^4^ Studies have reported that buprenorphine is not inferior to methadone on outcome measures assessing NAS and maternal and neonatal safety when treatment is initiated in the second trimester.^5,6,7^ Moreover, evidence suggests that buprenorphine confers additional benefits for neonates affected by NAS, including milder symptoms and shorter hospital lengths of stay, compared with neonates with in utero exposure to methadone.^5^

Buprenorphine is a cost-effective treatment for OUD in adults, prevents complications associated with nonfatal overdoses, reduces the risk of injection-related infections, and reduces mortality risk.^8,9,10,11^ Despite its effectiveness, buprenorphine remains inaccessible for many women with OUD who are pregnant. A study of treatment episodes for prescription OUD during pregnancy showed that medication for OUD was administered only during a third of treatment episodes,^1^ and a more recent study of women enrolled in Medicaid who were pregnant noted that nearly half of pregnant patients with OUD receive no medication for OUD.^12^

The low rate of pregnant women with OUD receiving evidence-based care is due, in part, to barriers physicians face in terms of the ability to prescribe the medication. The Drug Addiction Treatment Act requires 8 hours of training for physicians and 24 hours for nurse practitioners and physician assistants to receive approval via a waiver (hereafter called the X-waiver) by the Drug Enforcement Agency to prescribe buprenorphine.^13,14^ A recent study showed that only 0.4% of obstetrician-gynecologists were X-waivered.^15^ To our knowledge, despite increasing rates of OUD among women who are pregnant, no study has focused on the geographic distribution of X-waivers among obstetricians and gynecologists. The objective of this study, therefore, was to describe the geographic distribution and characteristics associated with X-waiver status among a broad sample of US obstetrician-gynecologists in relation to the severity of the opioid epidemic.

## Methods

### Study Design

This cross-sectional analysis linking physician-specific data to county- and state-level data across the US was conducted from September 1, 2019, to March 31, 2020. This study followed the Strengthening the Reporting of Observational Studies in Epidemiology (STROBE) reporting guideline for cross-sectional studies. Because this study used only publicly available data, it was considered exempt by the University of Pennsylvania institutional review board. The initial data sets had identifiers that allowed us to merge them appropriately but were deidentified for the purpose of the analysis.

Data for this study were acquired from 4 different sources. Individual physician data were acquired from the August 2019 Drug Enforcement Agency Substance Abuse and Mental Health Services Agency (SAMHSA) X-waivered practitioners data set^16^ and the September 2019 Center for Medicare & Medicaid Services (CMS) Physician Compare data set.^17^ SAMHSA provides a data set of all clinicians with an X-waiver permitting them to prescribe buprenorphine in the US states and territories. The CMS Physician Compare is a data set of all physicians who have filed at least 1 Medicaid claim in the previous 3 months. The CMS data identify the specialty of the physician.

County-level data were acquired by geocoding all physicians’ work addresses provided by the CMS Physician Compare data set in ArcGIS, version 10.0 (Esri) and merging the geocoded data with county socioeconomic data from the Robert Wood Johnson Foundation’s 2019 County Health Rankings and Roadmaps,^18^ which combines socioeconomic, demographic, and health statistics from corresponding US federal agencies’ publicly available data, as well as state-level NAS rates reported by the national inpatient sample data.^19^ The US Department of Agriculture Economic and Research Service’s rural-urban continuum was used to characterize counties as metropolitan or rural.^20^

We selected the CMS Physician Compare data set based on their listed primary specialty for obstetrics and gynecology and merged this data set with the SAMHSA data based on unique identifiers: first name, last name, and zip code were first used to identify matches between the 2 databases. Duplicates were eliminated by using National Provider Identifier Number as provided by the CMS Physician Compare data set.

### Baseline Covariates

Clinician-level baseline variables included sex, number of years since medical school graduation, and whether they have hospital privileges. We chose to compare individual physicians based on available variables with the aim of controlling for factors that may explain why some physicians are not X-waivered. In addition, if an obstetrician-gynecologist does not have hospital privileges, they likely do not work in labor and delivery settings, which is an important point of contact where women who are pregnant are increasingly diagnosed with OUD, as documented by the Centers for Disease Control and Prevention.^21^ Area-level variables used included county median household income, percentage of Black residents, percentage of Hispanic residents, rurality, percentage of uninsured residents, and most recently reported state NAS rate.

### Statistical Analysis

Descriptive statistics were used to describe X-waivered obstetrician-gynecologists and non–X-waivered obstetrician-gynecologists. A multivariable binomial logistic regression was used to determine independent associations with the presence or absence of X-waivered status. Analysis was conducted with Stata, version 16 (StataCorp LLC). Using paired 2-tailed *t* tests, statistical significance was defined as *P* < .05. Our model did not account for the nonrandomness of missing data.

## Results

In the CMS Physician Compare data set, 31 211 physicians were identified as being obstetrician-gynecologists as of September 13, 2019, representing approximately three-quarters of US obstetrician-gynecologists, according to the American Association of Medical Colleges’ most recent census of number of active physicians per specialty (2017)^22^ and the Doximity 2018 obstetrician-gynecologists workforce study.^23^

Table 1 reports the characteristics of the population. Among the obstetrician-gynecologists identified, 12 501 were male (40.1%) and 18 710 were female (59.9%). Most had hospital privileges (23 236 [74.4%]). The mean (SD) number of years since medical school graduation was 23.5 (12.0). Most (28 613 [91.7%]) worked in metropolitan counties and few (60 [0.2%]) worked in entirely rural counties.

**Table 1.** Demographic and Area-Level Characteristics of Obstetrician-Gynecologists Who Accept Medicaid Based on Buprenorphine-Approved Status

Characteristic	Obstetrician-gynecologists, No. (%)		*P* value
	Non–X-waivered (n = 30 651)^a^	X-waivered (n = 560)^a^	
Individual level			
Male (n = 12 501)	12 236 (39.9)	265 (47.3)	<.001
Female (n = 18 710)	18 415 (60.1)	295 (52.7)	
No hospital privileges	7828 (25.5)	147 (26.3)	.07
Years since medical school graduation, No.			
0-10	4372 (14.3)	84 (15.0)	.04
11-20	8214 (26.8)	160 (28.6)	
21-30	8460 (27.6)	154 (27.5)	
31-40	6373 (20.8)	116 (20.7)	
>40	3232 (10.5)	46 (8.2)	.15
County level, mean (SD)			
Median household income, $	64 786 (17 550.8)	59 564 (18 056.4)	<.001
% Uninsured residents	9.50 (0.03)	8.82 (0.16)	<.001
% Non-Hispanic Black residents	14.28 (13.01)	13.23 (12.98)	.03
% Hispanic residents	17.27 (15.91)	12.83 (13.23)	<.001
Rural-urban continuum			
Counties in metropolitan areas with population ≥1 million	18 507 (60.4)	291 (52.0)	<.001
Counties in metropolitan areas with population 250 000 to 1 million	6803 (22.2)	141 (25.2)	
Counties in metropolitan areas with population <250 000	2803 (9.2)	48 (8.6)	
Urban population ≥20 000, adjacent to a metropolitan area	928 (3.0)	32 (5.7)	
Urban population ≥20 000, not adjacent to a metropolitan area	509 (1.7)	12 (2.1)	
Urban population 2500-19 999, adjacent to a metropolitan area	554 (1.8)	19 (3.4)	
Urban population 2500-19 999, not adjacent to a metropolitan area	468 (1.5)	15 (2.7)	
Completely rural or population ≤2500	58 (0.2)	2 (0.4)	
State level			
State NAS rate, mean (SD)	7.645 (5.93)	10.62 (7.53)	<.001
0-5 per 1000 births	11 637 (38.0)	97 (17.3)	<.001
5-10 per 1000 births	10 309 (33.6)	182 (32.5)	
10-15 per 1000 births	6112 (19.9)	190 (33.9)	
>15 per 1000 births	1648 (5.4)	77 (13.8)	
NA	945 (3.1)	14 (2.5)	

Abbreviations: NA, not available; NAS, neonatal abstinence syndrome.

^a^ X-waivered indicates obstetrician-gynecologists who are trained to prescribe buprenorphine.

**Figure 1.**
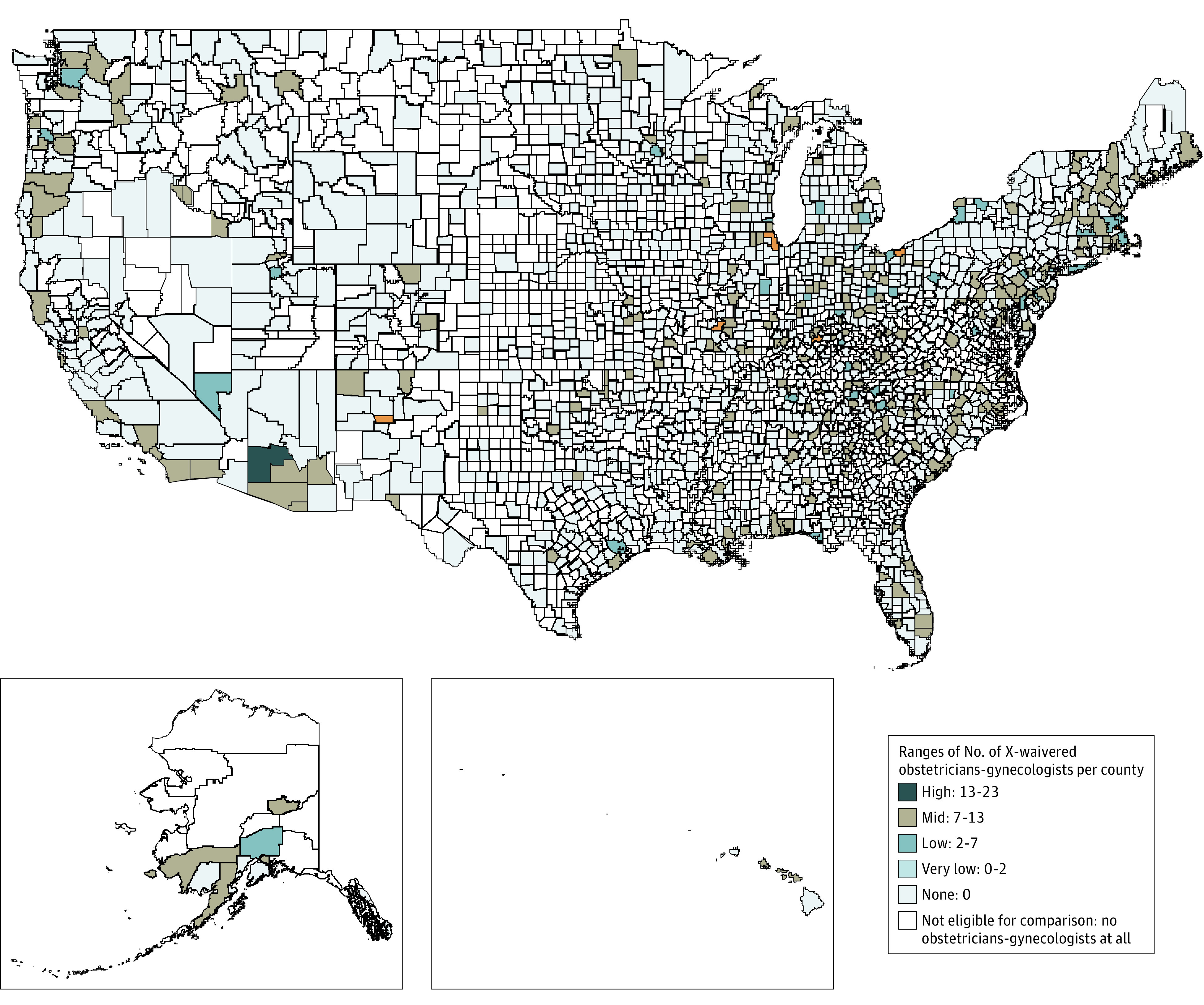
Distribution of Obstetrician-Gynecologists Who Can Prescribe Buprenorphine by US Counties With at Least 1 Medicaid-Claimant Obstetrician-Gynecologist

Linking this data set with the SAMHSA data set of buprenorphine-approved physicians yielded a total of 560 obstetrician-gynecologists (1.8%) who were X-waivered. Figure 1 provides a geographic distribution of X-waivered obstetrician-gynecologists by county with at least 1 Medicaid-claimant obstetrician-gynecologist. Male obstetrician-gynecologists were slightly more likely to be X-waivered (265 of 12 501 [2.1%]) compared with their female counterparts (295 of 18 710 [1.6%]) (*P* < .001). X-waivered obstetrician-gynecologists had fewer mean (SD) years since medical school graduation (23.5 [11.5] years) compared with their non–X-waivered counterparts (24.5 [12.0] years) (*P* < .001).

**Figure 2.**
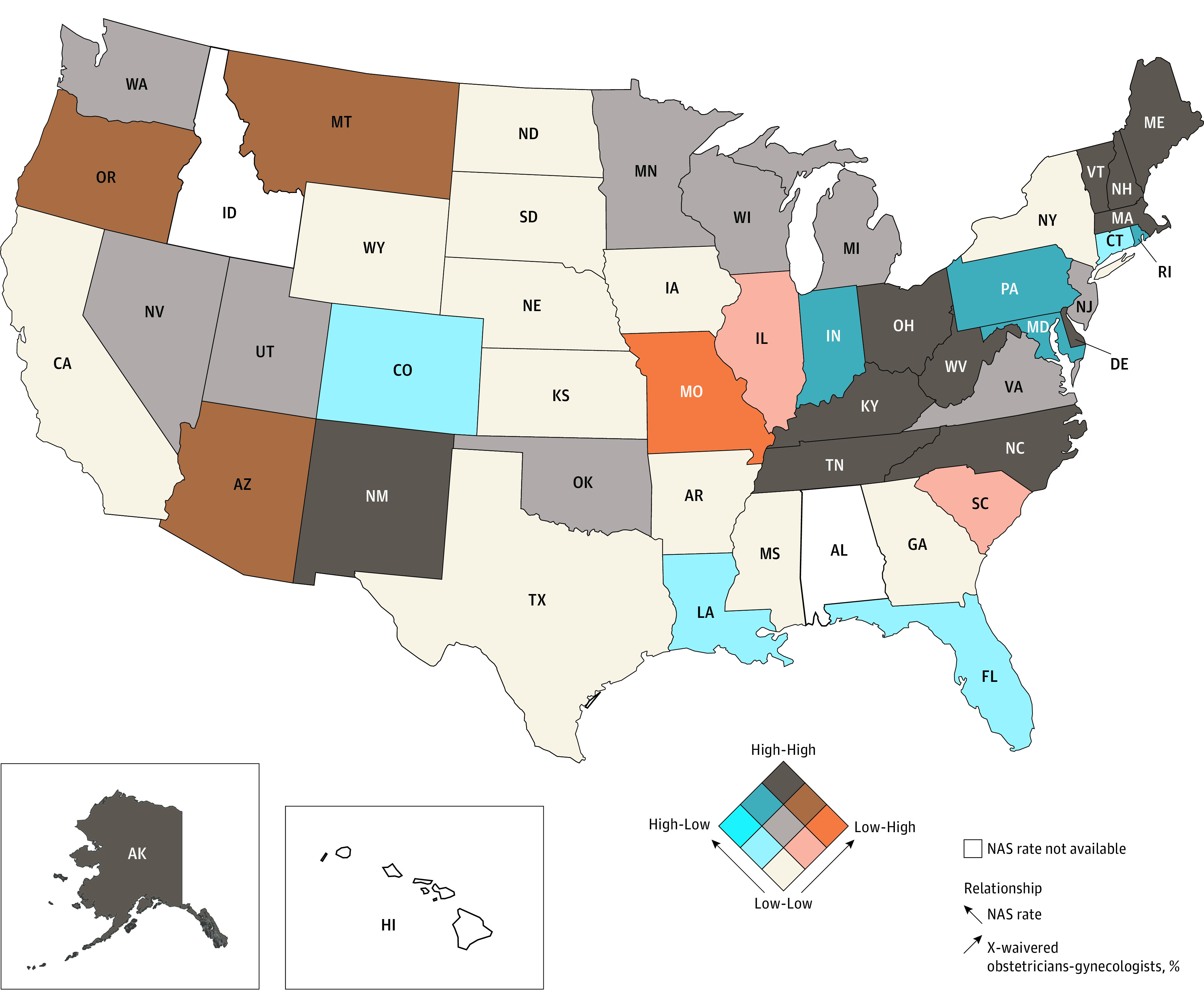
Geographic Distribution of Obstetrician-Gynecologists Who Can Prescribe Buprenorphine in Relation to the Neonatal Abstinence Syndrome (NAS) Rate Percentages of obstetrician-gynecologists who can prescribe buprenorphine were classified into the following tertiles: 0% to 1.0% (low), 1.0% to 2.6% (medium), and 2.6% to 9.0% (high). A state’s NAS rate was classified in the following tertiles: 1.0 to 5.0 per 1000 births (low), 5.0 to 9.6 per 1000 births (medium), and 9.6 to 56.0 per 1000 births (high).

In the multivariate logistic regression (model 1), X-waivered status was associated with male sex (adjusted odds ratio [aOR], 1.52; 95% CI, 1.26-1.83; *P* < .001) and not having hospital privileges (aOR, 1.32; 95% CI, 1.08-1.61; *P* = .007) (Table 2). The state NAS rate was also associated with X-waivered status (Figure 2). For example, compared with an NAS rate ranging from 0 to 5 per 1000 births, obstetrician-gynecologists in states with NAS rates greater than 15 per 1000 births had nearly 5 times the odds of being X-waivered (aOR, 4.94; 95% CI, 3.60-6.77; *P* < .001). Obstetrician-gynecologists in counties with less than 5% uninsured residents had nearly twice the odds as those in counties with greater than 15% uninsured residents to be X-waivered (aOR, 1.59; 95% CI, 1.04-2.44; *P* = .04).

**Table 2.** Model Estimating Odds of Medicaid-Claimant Obstetrician-Gynecologists X-Waivered Status^a^

Dependent variable: X-waivered status	Model 1 (n = 30 251)		Model 2 (states’ fixed effects, n = 30 078)	
	aOR (95% CI)	*P* value	aOR (95% CI)	*P* value
Sex				
Female	1 [Reference]		1 [Reference]	
Male	1.52 (1.26-1.83)	<.001	1.55 (1.28-1.87)	<.001
Hospital privileges				
Yes	1 [Reference]		1 [Reference]	
No	1.32 (1.08-1.61)	.007	1.33 (1.08-1.63)	.007
No. of years since medical school graduation				
>40	1 [Reference]		1 [Reference]	
0-10	1.61 (1.09-2.38)	.02	1.59 (1.09-2.34)	.02
11-20	1.67 (1.17-2.38)	.005	1.61 (1.13-2.28)	.008
21-30	1.47 (1.04-2.07)	.03	1.39 (0.99-1.96)	.06
31-40	1.29 (0.91-1.84)	.16	1.25 (0.88-1.78)	.20
Rural-urban continuum				
Counties in metropolitan areas with population ≥1 million	1 [Reference]		1 [Reference]	
Counties in metropolitan areas with population 250 000 to 1 million	1.15 (0.93-1.42)	.19	1.20 (0.96-1.51)	.12
Counties in metropolitan areas with population <250 000	0.94 (0.68-1.31)	.72	0.95 (0.68-1.325)	.76
Urban population ≥20 000, adjacent to a metropolitan area	1.85 (1.26-2.71)	.002	1.64 (1.11-2.43)	.01
Urban population ≥20 000, not adjacent to a metropolitan area	1.48 (0.79-2.74)	.22	1.37 (0.73-2.58)	.33
Urban population 2500-19 999, adjacent to a metropolitan area	1.68 (1.01-2.79)	.05	1.76 (1.08-2.87)	.02
Urban population 2500-19 999, not adjacent to a metropolitan area	1.88 (1.09-3.24)	.02	1.69 (0.96-2.95)	.07
Completely rural or population ≤2500	2.57 (0.61-10.83)	.20	1.81 (0.42-7.78)	.42
% Uninsured^b^				
>15	1 [Reference]			
0-5	1.59 (1.04-2.44)	.04		
5-10	1.49 (1.03-2.16)	.04		
10-15	1.58 (1.08-2.31)	.02		
NAS rate^b^				
0-5 per 1000 births	1 [Reference]			
5-10 per 1000 births	2.04 (1.59-2.62)	<.001		
10-15 per 1000 births	3.51 (2.71-4.53)	<.001		
>15 per 1000 births	4.94 (3.60-6.77)	<.001		

Abbreviations: aOR, adjusted odds ratio; NAS, neonatal abstinence syndrome.

^a^ X-waivered indicates obstetrician-gynecologists who are trained to prescribe buprenorphine.

^b^ Excluded from model 2 owing to collinearity.

Compared with those located in large metropolitan areas (counties in metropolitan areas of ≥1 million people), obstetrician-gynecologists in suburban counties (eg, urban population ≥20 000 and adjacent to a metropolitan area) were more likely to be X-waivered (aOR, 1.85; 95% CI, 1.26-2.71; *P* = .002). Obstetrician-gynecologists without hospital privileges were more likely to be X-waivered (aOR, 1.32; 95% CI, 1.08-1.61; *P* = .007). We performed a second multivariable regression with states’ fixed effects (excluding NAS rates and percentage of uninsured rates owing to collinearity) to account for differences in state-level policies and the landscape of the opioid epidemic. In this model, male obstetrician-gynecologists were also more likely to be X-waivered (aOR, 1.55; 95% CI, 1.28-1.87; *P* < .001), as were those without hospital privileges (aOR, 1.33; 95% CI, 1.08-1.63; *P* = .007).

To check for the robustness of these models, we performed logistic regressions with number of years since medical school graduation, NAS rate, and percentage of uninsured individuals as continuous variables reported as aORs for every increase by SD. The findings remained statistically significant. In both models, the odds of being X-waivered decreased by 14% (95% CI, 5%-22%) with each increase in SD of number of years since medical school graduation (eTable 1 in the Supplement). eTable 2 in the Supplement includes variance inflation factors for each model, accounting for multicollinearity between covariates.

## Discussion

This study had 4 main findings. First, fewer than 2% of obstetrician-gynecologists who treat patients with Medicaid insurance are X-waivered to prescribe buprenorphine. Second, obstetrician-gynecologists who are X-waivered were more likely to work in suburban counties than large metropolitan counties and more likely to work in counties with lower rates of uninsured residents. Third, obstetrician-gynecologists who are X-waivered were less likely to have hospital privileges and thus primarily work in outpatient settings, and more likely to be male and to have graduated from medical school more recently. Fourth, obstetrician-gynecologists who are X-waivered were more likely to be located in states with relatively high NAS rates. These findings highlight gaps and opportunities for improving the role that obstetrician-gynecologists could play in addressing the opioid epidemic by increasing the capacity of physicians capable of providing evidence-based treatment for OUD.

This study adds to current evidence on the geographic distribution and prevalence of X-waivered physicians and disparities in access to buprenorphine. The low percentage of obstetrician-gynecologists who are able to prescribe buprenorphine reflects prior studies’ findings on the overall distribution of physicians with X-waivers. A previous study using the American Medical Association master file including non-Medicaid claimant physicians reported that 2.2% of US physicians had obtained X-waivers. Among those physicians, only 1% were obstetrician-gynecologists. The same study noted that 0.4% of obstetrician-gynecologists were X-waivered.^24,25^ Given that X-waivered status in our study was associated with greater state-level NAS rates, it is possible that the relative increase in the proportion of obstetrician-gynecologists who are X-waivered occurred in response to the progression of the opioid epidemic. A recent study of NAS rates and treatment access showed a correlation between NAS rates and opioid prescribing rates in states with greater density of X-waivered physicians. Another explanation for this correlation is that in utero exposure to buprenorphine can lead to NAS.^26^

We found a higher prevalence of X-waivered obstetrician-gynecologists in suburban counties and in counties with fewer uninsured residents. This finding aligns with prior evidence that, despite overall uptake in buprenorphine use,^27^ access remains uneven based on race, insurance status, and geographic area.^28^ As demonstrated by a recent national study, buprenorphine treatment is concentrated among White patients and those with private insurance.^29^ Studies have shown that, as a result of racial segregation, buprenorphine availability is associated with a greater proportion of White residents at the neighborhood and county levels, and methadone availability is associated with greater proportions of Hispanic and Black residents.^30,31^ Previous studies described substantial state- and county-level imbalances in the availability of buprenorphine practitioners, and OUD treatment capacity and the burden of opioid overdose deaths within the US.^32,33^ In terms of rurality, previous studies noted that X-waivered physicians working in rural counties were more likely to be primary care physicians.^34^ In addition, nurse practitioners and physician assistants accounted for more than half of X-waivered clinicians in rural counties between 2016 and 2019.^35^

Within the context of pregnancy, a study reported that Black and Hispanic women (both overrepresented among Medicaid recipients) are less likely to receive any pharmacotherapy for OUD. When receiving pharmacotherapy, Black and Hispanic women are more likely to receive methadone than buprenorphine,^12^ and those receiving buprenorphine are more likely to be married, be employed, and have more education.^36^ Although questions of continued adherence to buprenorphine compared with methadone are often raised,^37^ it has also been shown that pregnant women who are transitioned from methadone to buprenorphine have comparable outcomes to those who had received buprenorphine throughout their pregnancy.^38^ Methadone-based treatment has been described as rigid owing to the required daily visits; however, buprenorphine affords patients additional flexibility and privacy. For women with OUD who are pregnant, having the option of being treated with buprenorphine could thus reduce the burden of increased clinic visits during pregnancy and thereafter should they continue to receive buprenorphine.^39,40^

Concerns of retention in treatment, especially in the postpartum phase, may be addressed by initiation of the buprenorphine extended-release formulation. In the general population, patients receiving buprenorphine extended-release report higher treatment satisfaction, and the medication leads to greater positive, patient-centered outcomes.^41^ These benefits are particularly important in the postpartum period, a challenging time in a woman’s life with drug overdoses being a leading cause of death.^42^ Use of buprenorphine extended-release, however, requires that patients have a history of receiving the immediate-release formulation. Evidence suggests that women with OUD who are pregnant in states most affected by the opioid epidemic face substantial barriers accessing both buprenorphine and methadone. Methadone practitioners are more likely than X-waivered clinicians to accept women who are pregnant, but rates of accepting pregnant women in both groups are low. In addition, Medicaid is accepted at low rates overall, and X-waivered clinicians accept cash payments at higher rates.^43,44^

The observed sex difference in the rates of X-waivered obstetrician-gynecologists in our study may be reflective of prior evidence that showed differences in practice styles, whereby male obstetrician-gynecologists were more likely to manage the nonreproductive health conditions of their patients, while female obstetrician-gynecologists were more likely to refer the patients to primary care physicians or other specialists.^45^

The finding that obstetrician-gynecologists with hospital privileges are less likely to be X-waivered is in line with a recent study showing that, overall, X-waivered physicians are most likely to have primarily outpatient practices and not be integrated with hospital systems.^46^ This lack of integration presents an opportunity for improving continuity and transitions in care, especially after key events such as the transition from birthing to outpatient postpartum care.

The overall low number of obstetrician-gynecologists who are X-waivered may be associated with several factors, including existing barriers, stigma, and historical precedent. Historically, X-waivered physicians are more likely to be psychiatrists and primary care physicians. However, other specialties have seen significant increases in X-waiver status, including emergency medicine.^15,24,25^ Even among women with OUD who are pregnant and enrolled in Medicaid, primary care physicians and psychiatrists are the leading prescribers of buprenorphine, followed by obstetrician-gynecologists.^47^ Clinicians face barriers to becoming X-waivered, including low psychosocial support, time constraints, and stigma from colleagues and office staff, which altogether may also contribute to this low rate.^48,49,50^ Some obstetrician-gynecologists who are not X-waivered may work in collaboration with psychiatrists or primary care physicians to whom they refer their patients for buprenorphine, but studies show that women who are pregnant are unevenly screened and referred to treatment by their obstetrician-gynecologists after screening positive for substance use disorders,^51^ despite what guidelines recommend. Initiation of treatment after positive screening and bridging to long-term care is a useful practice to retain patients in treatment and is an increasing practice in emergency department settings.^52,53,54^ Screening can take place during prenatal care, as recommended by the American College of Obstetrics and Gynecology,^4^ or at delivery, in addition to newborn NAS screening. Not being X-waivered limits physicians’ ability to initiate and bridge patients to long-term treatments in case of a positive screening and patient interest in initiation of medications. These barriers, furthermore, contribute to racial/ethnic and socioeconomic disparities in access to buprenorphine.^29,55^

In terms of the association between NAS rates and the distribution of X-waivered obstetrician-gynecologists, our findings suggest the prevalence of X-waivered obstetrician-gynecologists tracks with NAS rates, although absolute numbers are low. Some states, however (eg, Florida, Colorado, Connecticut, and Louisiana), show a relative lag, where NAS rates are relatively high, while the prevalence of X-waivered obstetrician-gynecologists is relatively low compared with other states. This relative lag may be due to local policies, such as states’ responses to the opioid epidemic. Regional socioeconomic factors contribute to NAS rates^56^ but may not necessarily influence adoption of the X-waiver at the same rate.

Beyond pregnancy, there remains a role for X-waivered obstetrician-gynecologists. A study evaluating the reproductive needs of clients with substance use disorder noted that 83% of women and 58% of men would use family planning services if available at their place of treatment. In addition, only 53% of sexually active women reported using any form of contraception, and 20% reported using a highly reliable form of contraception.^57^ Given the increasing prevalence of OUD among women, and as substance use disorder moves toward greater integration with health services provision, X-waivered obstetrician-gynecologists could fill a gap in OUD care, family planning, and general gynecologic care for women with OUD. Our findings warrant further investigation into barriers that obstetrician-gynecologists face with regard to becoming X-waivered.

### Limitations

Our study has limitations. Data were limited to obstetrician-gynecologists who treat patients enrolled in Medicaid and therefore do not include all US obstetrician-gynecologists. However, nearly 50% of births in the US are paid for by Medicaid,^58^ and over 80% of newborns with NAS are insured by Medicaid.^59^ A recent study reported that X-waivered physicians prescribe buprenorphine at varying rates, including some who do not prescribe buprenorphine.^60^ Our findings may thus not reflect the degree to which X-waivered obstetrician-gynecologists may be prescribing buprenorphine. In addition, NAS data for 2 states (Idaho and Alabama) were not available, which we did not account for in our analysis.

## Conclusions

This study found that only 1.8% of Medicaid-claimant obstetrician-gynecologists are approved to prescribe buprenorphine. Our study contributes to the body of work describing the geographic distribution of health care clinicians who are able to prescribe buprenorphine. Given the relative benefits that buprenorphine confers toward maternal and neonatal health compared with methadone or no pharmacotherapy, as well as existing racial/ethnic and socioeconomic disparities in access to buprenorphine, these findings suggest that obstetrician-gynecologists may play a greater role in addressing the opioid epidemic beyond referring patients to OUD treatment. By becoming X-waivered, obstetrician-gynecologists could bolster the workforce of health care clinicians authorized to initiate and maintain OUD treatment.

## Notes

Supplement. **eTable 1.** Robustness Check, Models Estimating Odds of Medicaid Claimant Obstetrician-Gynecologist X-Waivered Status **eTable 2.** Variance Inflation Factors for All Logistic Regression Models
