# Supplementary material for: Prevalence and Geographic Distribution of Obstetrician-Gynecologists Who Treat Medicaid Enrollees and Are Trained to Prescribe Buprenorphine
Source: JAMA Netw Open. 2020 Dec 11;3(12):e2029043. doi: 10.1001/jamanetworkopen.2020.29043 (PMC7733157; doi:10.1001/jamanetworkopen.2020.29043)
Supplement: Supplement. — eTable 1. Robustness Check, Models Estimating Odds of Medicaid Claimant Obstetrician-Gynecologist X-Waivered Status eTable 2. Variance Inflation Factors for All Logistic Regression Models [file jamanetwopen-e2029043-s001.pdf]

Supplementary Online Content

Nguemeni Tiako MJ, Culhane J, South E, Srinivas SK, Meisel ZF. Prevalence and geographic distribution of obstetrician-gynecologists who treat Medicaid enrollees and are trained to prescribe buprenorphine. *JAMA Netw Open*. 2020;3(12):e2029043. doi:10.1001/jamanetworkopen.2020.29043

eTable 1. Robustness Check, Models Estimating Odds Of Medicaid Claimant Obstetrician-Gynecologist X-Waivered Status

eTable 2. Variance Inflation Factors for All Logistic Regression Models

This supplementary material has been provided by the authors to give readers additional information about their work.

© 2020 Nguemeni Tiako MJ et al. *JAMA Network Open.*

**eTable 1:** Robustness check, Models estimating odds of Medicaid claimant Obstetrician-Gynecologist X-waivered status				
	Model 1 (n = 30,251)		Model 2 (states fixed effects) (n = 30,078)	
	aOR (95% CI)	P-value	aOR (95% CI)	P-value
**Sex**				
Female	1 (ref)			
Male	**1.53 (1.26-1.85)**	**<0.001**	1.56 (1.29-1.89)	<0.001
**Hospital privileges**				
Yes	1 (ref)			
No	**1.31 (1.07-1.60)**	**0.008**	**1.32 (1.07-1.62)**	**0.008**
**Number of years since medical school graduation, per standard deviation**	**0.86 (0.78-0.95)**	**0.002**	**0.86 (0.78-0.95)**	**0.002**
**Rural Urban Continuum**				
Counties in metro areas of 1 million population or more	1 (ref)		1 (ref)	
Counties in metro areas of 250,000 to 1 million population	1.16 (0.94-1.43)	0.18	1.20 (0.95-1.50)	0.12
Counties in metro areas of fewer than 250,000 population	0.92 (0.67-1.28)	0.63	0.95 (0.68-1.32)	0.75
Urban population of 20,000 or more, adjacent to a metro area	**1.86 (1.27-2.72)**	**0.001**	**1.64 (1.11-2.42)**	**0.01**
Urban population of 20,000 or more, not adjacent to a metro area	1.39 (0.75-2.58)	0.29	1.36 (0.72-2.57)	0.34
Urban population of 2,500 to 19,999, adjacent to a metro area	1.65 (0.99-2.73)	0.05	**1.75 (1.08-2.86)**	**0.02**
Urban population of 2,500 to 19,999, not adjacent to a metro area	**1.73 (1.01-2.97)**	**0.05**	1.68 (0.96-2.96)	0.07
Completely rural or with a population of 2,500 or less	2.26 (0.54-9.49)	0.26	1.80 (0.42-7.70)	0.43
**% Uninsured per standard deviation**	0.92 (0.83-1.01)	0.1		-
**State NAS rate per standard deviation**	**1.65 (1.52-1.79)**	**<0.001**		

© 2020 Nguemeni Tiako MJ et al. *JAMA Network Open.*

**eTable 2**: Variance Inflation factors for all logistic regression models				
	Model 1	Model 2	Robustness Check Model 1	Robustness check model 2
Sex				
Female	ref			
Male	1.73	1.99	2.05	2.09
Hospital privileges				
Yes	ref			
No	1.36	1.50	1.36	1.5
**Number of years since medical school graduation**				
>40 years	Ref			
0-10	1.75	2.46		
10-20years	2.34	3.64		
20-30years	2.31	3.52		
30-40years	2.04	2.8		
Years per SD			4.89	6.13
**Percent Uninsured**				
>15%	ref			
0-5%	1.79		-	-
5-10%	3.71		-	-
10-15%	2.50		-	-
**% Uninsured per standard deviation**			3.71	-
**Rural Urban Continuum**				
Counties in metro areas of 1 million population or more	Ref			
Counties in metro areas of 250,000 to 1 million population	1.39	1.63	1.38	1.61
Counties in metro areas of fewer than 250,000 population	1.17	1.28	1.16	1.27
Urban population of 20,000 or more, adjacent to a metro area	1.07	1.11	1.07	1.11
Urban population of 20,000 or more, not adjacent to a metro area	1.04	1.14	1.04	1.14
Urban population of 2,500 to 19,999, adjacent to a metro area	1.04	1.07	1.04	1.06
Urban population of 2,500 to 19,999, not adjacent to a metro area	1.04	1.09	1.04	1.08
Completely rural or with a population of 2,500 or less	1.01	1.02	1.01	1.01

© 2020 Nguemeni Tiako MJ et al. *JAMA Network Open.*

**NAS rate**				
0-5/1000 births	Ref			
5-10/1000 births	1.90	-	-	-
10-15/1000 births	1.68	-	-	-
>15/1000 births	1.21	-	-	-
**State NAS rate per standard deviation**	-	-	3.53	-

© 2020 Nguemeni Tiako MJ et al. *JAMA Network Open.*
